# Effectiveness of Mouthwash, Rinses, Salt Water, and Toothpastes on the Oral Health of Pregnant Women: A Systematic Review: Pregnancy and Oral Health

**DOI:** 10.1155/ghe3/2627524

**Published:** 2026-05-25

**Authors:** Muhammad Mohsin Javaid, Mariyum Sarfraz, Khola Noreen, Ume Habiba Johar, Muhammad Mutahir Mehdi, Najam Ul Hassan, Shumaila Zofeen, Shazia Iqbal, Shahzad Ahmad, Muhammad Farooq Umer

**Affiliations:** ^1^ School of Public Health, Health Services Academy, Islamabad, Pakistan, hsa.edu.pk; ^2^ Department of Community Medicine, Rawalpindi Medical University, Rawalpindi, Pakistan; ^3^ Department of Oral Biology, Nishtar Institute of Dentistry, Multan, Pakistan; ^4^ Department of Orthodontics and Dentofacial Orthopedics, School of Dentistry, Shaheed Zulfiqar Ali Bhutto Medical University, Islamabad, Pakistan, szabmu.edu.pk; ^5^ School of Public Health, Xi’an Jiaotong University, Xi’an, Shaanxi, China, xjtu.edu.cn; ^6^ Faculty of Medicine and Health Sciences, The University of Buckingham, Buckingham, UK, upm.edu.my; ^7^ IMBB, The University of Lahore, Lahore, Pakistan, uol.edu.pk; ^8^ Department of Preventive Dental Sciences, College of Dentistry, King Faisal University, Hofuf, 31982, Al Ahsa, Saudi Arabia, kfu.edu.sa

**Keywords:** mouthwash, oral health, pregnancy, saltwater rinse, toothpaste

## Abstract

**Introduction:**

Pregnancy is linked to hormonal fluctuations, making pregnant women more susceptible to gingival and periodontal diseases. Hence, oral hygiene interventions, such as mouthwashes and toothpastes, play a crucial role in maintaining oral hygiene among women. However, the interventions used should be effective as well as safe to use. Hence, this systematic review assesses the effectiveness of various oral hygiene interventions, which include mouthwashes, saltwater rinses, and toothpastes, on the oral health of pregnant women.

**Methods:**

A detailed literature search was done using PubMed, Web of Science, ScienceDirect, and Google Scholar, including studies published in the last 6 years. Only randomized controlled trials, clinical trials, and systematic reviews relevant to the objective of this study were included. The quality assessment of the included studies was done using the Joanna Briggs Institute (JBI) critical appraisal tool. According to this tool, all the selected studies had moderate‐to‐high quality.

**Results:**

The three studies were finally selected that met the inclusion/exclusion criteria based on the PRISMA flowchart. Alcohol‐free mouthwashes including fluorides, antibacterial toothpastes, and saltwater rinses were found to improve gingival health and decrease plaque accumulation and gingival inflammation. Some additional agents were found to be as effective as chlorhexidine during pregnancy.

**Conclusion:**

Oral hygiene interventions based on saltwater rinses, fluoridated mouthwashes, and toothpastes improve oral health in pregnant women. These agents are effective and easily available for better oral hygiene during pregnancy and better pregnancy outcomes.

## 1. Introduction

Periodontal diseases not only affect the normal human body but also have extremely adverse effects on those with various systemic diseases or conditions, including pregnancy [1]. Along with the association of periodontal diseases with various systemic diseases, their adverse effects on pregnancy outcomes have been noted. Henceforth, special populations require proper screening and care for periodontal health [2]. According to the study carried out in Samarkand, the prevalence of caries and other dental diseases was found to be 70%–95% among pregnant women, which was due to a lack of knowledge and awareness [3]. Moreover, higher microflora levels were noted in the oral cavity during pregnancy, regardless of how extreme dental care can reduce these oral microflorae (e.g., *Streptococcus mutans*) [4]. Oral health interventions are of the utmost importance during pregnancy since most dental procedures are deferred during this period [5]. Overall, developing countries like Iran have adequate knowledge, a positive attitude, and good practices regarding oral health among pregnant women [6]. Daily mouthwash brushing using a dentifrice containing herbal ingredients and zinc had better effects in plaque control and improved gingival health in comparison to that of fluoride‐containing toothpaste [7]. Interventional programs involving oral health awareness by oral healthcare workers and trained professionals before and after pregnancy can greatly impact the oral health status and practices of pregnant women and lactating mothers [8]. One more 6‐week double‐blind randomized controlled trial (RCT) was also conducted in 2024, in which zinc‐containing toothpastes were used by the participants for around 6 weeks. Significant improvements in the plaque control and gingival health of the recruited participants were observed, signifying better results. Although it was not conducted among pregnant women, the safety and efficacy of zinc formulations make it inclusive for all [9]. Toothpastes, shampoo, and deodorants are the most common cosmetic and hygiene products used by pregnant women [10]. Moreover, one of the studies concluded that toothpastes are a daily‐use product, and not a luxury, during pregnancy as well as postpartum [11]. The most studied chemical agent in the oral cavity is chlorhexidine, followed by cetylpyridinium chloride (CPC) and fluorides. Licorice herb and curcumin also possessed the maximum efficacy. The presence of plaque‐controlling agents in toothpastes and mouthwashes helps prevent plaque accumulation and periodontal diseases. However, this study did not explain the consequences of these products in the pregnant population [12]. In addition, sodium chloride possesses the same anti‐inflammatory properties as chlorhexidine mouth rinse, so it can be used regularly after periodontal surgeries. Mouthwashes containing saltwater had an anti‐inflammatory effect similar to that of 0.12% chlorhexidine just after minimally invasive periodontal surgery. Moreover, saltwater mouthwash is readily available worldwide. However, this study also did not include pregnant women [13].

As far as we know, there is very little literature on oral health interventions among pregnant women. Therefore, this systematic review, conducted among pregnant women, aimed to evaluate the effectiveness of various oral health interventions (including mouthwashes, salt rinses, and toothpastes) on their oral health. This will also aid future researchers in conducting research on the given topic. This systematic review is based on Preferred Reporting Items for Systematic Reviews and Meta‐Analyses (PRISMA) guidelines.

## 2. Methodology

### 2.1. Search Strategy

To build a search strategy, three databases were selected to review the literature using the international PRISMA guidelines [14]. A detailed literature review was conducted from three international databases, PubMed, Web of Science, and ScienceDirect, and gray literature was retrieved from Google Scholar. These search engines were selected as they are open‐access journals and easily accessible to the reviewers. Moreover, those articles published in the last 6 years were included in this study to ensure that the latest published literature is included that reflects the recent advancements made in the effectiveness of mouthwashes, rinses, and toothpastes for the oral health of pregnant women. The MeSH terms were used when searching PubMed using Boolean operators (AND, OR, and NOT). In contrast, keywords were used to retrieve relevant data from the Web of Science and ScienceDirect. The keywords used were “effects,” “mouthwashes,” “oral health conditions,” “pregnancy outcomes,” “pregnant women,” “rinses,” and “salt water,” The filters were applied to each search engine to include studies in the English language from the past 6 years only. Moreover, only clinical trials, RCTs, original articles, and systematic reviews were included. Additional filters of PubMed, like sex including females and age of more than 19 years, were also applied. The keywords used for search purposes on Web of Science and ScienceDirect were “mouthwashes,” “salt water rinses,” “chlorhexidine mouthwashes,” “oral health‐related quality of life,” “pregnancy outcomes,” and “pregnant women.” The filters applied for the Web of Science included social and behavioral sciences and life sciences from current contents.

### 2.2. Inclusion and Exclusion Criteria

Studies that addressed the pregnancy outcomes on oral health due to the use of mouthwashes, rinses, and saltwater were included through the search engines mentioned in the search strategy. Also, articles from the past 6 years in the English language were included in the study, and articles that were not in the English language, short communications, letters to the editor, or book chapters were excluded from the study. Studies with incomplete information were also excluded.

### 2.3. Selection of Studies

The procedure of selection of studies was done in two phases by the authors, M.M.J., U.H, K.N., N.U.H., and M.M.M. During each phase, if any disagreement arose between the two primary investigators, the other investigators, M.S., S.A., and M.F.U., were approached.

During the first phase, the authors reviewed the titles/abstracts of all the included articles and considered and reviewed them. After the removal of duplicates from all the above‐mentioned search engines, 330 articles were retrieved. At that point, the articles with incomplete data, including comorbidities, case reports/series, short communications, letters to the editors, and book chapters from the study, were removed, and only 36 articles were included.

**FIGURE 1:**
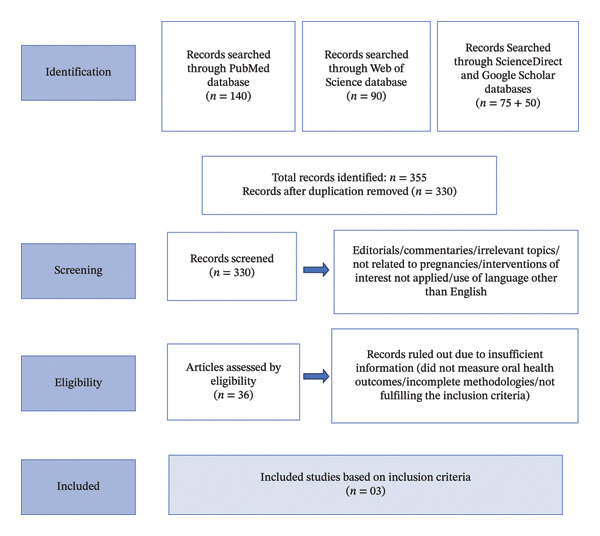
PRISMA flow diagram illustrating the selection of 03 studies.

During the second phase, the articles that included full texts were reviewed and included, and eventually, 03 articles were selected for inclusion in the present systematic review, which includes the effects of various mouthwashes, rinses, and saltwater on the oral health conditions of pregnant women in accordance with diagnostic accuracy (STARD). The flow diagram of the retrieved articles is mentioned below as Figure 1. The PRISMA flow diagram illustrates the entire procedure starting from the identification of records till the selection of included studies.

### 2.4. Data Abstraction

Following the online literature review, titles and abstracts of the studies were considered to extract the required data. Later, full texts were reviewed in detail to be included in the study. The data were extracted manually from the 03 studies that were finally selected. Using a predesigned form, authors(s), year, and country of the articles, study design, title, and the study outcomes were documented. These findings are mentioned in the results (Table 1). Table 1 illustrates the summary of results of studies included in this systematic review.

**TABLE 1:** Summary of results of studies included in the systematic review.

S. no.	Title of study	Type of study	Outcome of paper	Author (year/country)
1	Effectiveness of Alcohol‐Free Mouth Rinse Containing Essential Oils and Fluoride as an Oral Hygiene Adjunct Among Pregnant Thai Women: A Randomized Clinical Trial	A randomized clinical trial	There was no significant difference in the effects of alcohol‐free mouthwash containing essential oils with 0.05% fluoride content and alcohol‐free mouthwash with 0.05% fluoride only at the baseline period, 2 weeks, and 3 months postbaseline while measuring the oral health by self‐administered questionnaires and oral examination of plaque index, gingival status, and tongue cleanliness.	Hunsrisakhun et al. (2020/Thailand)
2	Evaluation of an Advanced Oral Hygiene Regimen on Maternity Outcomes in a Randomized Multicenter Clinical Trial (Oral Hygiene and Maternity Outcomes Multicenter Study)	A randomized controlled trial	The use of antibacterial toothpaste and nonalcoholic antimicrobial mouthwash in pregnant women improves oral hygiene by reducing the complications associated with gingivitis and preterm birth. It also provided insights into the safe use of common agents like cetylpyridinium chloride (CPC) and fluoride, being helpful in providing clinical care during pregnancy.	Parry et al. (2023/Birmingham, UK)
3	Clinical Use of Paraprobiotics for Pregnant Women with Periodontitis: Randomized Clinical Trial	A randomized clinical trial	Bleeding on probing and plaque control was significantly reduced when the paraprobiotic‐based toothpaste was used in combination with the paraprobiotic‐based mousse among the pregnant women. The recession process was also seen to be reduced. However, no significant changes were noted when the paraprobiotic‐based toothpaste was used alone.	Butera et al. (2024/Italy)

## 3. Results

### 3.1. Quality Assessment

The Joanna Briggs Institute (JBI) critical appraisal tool was employed to evaluate the quality of the included studies. This tool examined the randomization procedure used in the studies, the blinding techniques applied, the comparability of the groups, the follow‐up of the recruited samples, and the appropriateness of the statistical analysis. The studies fulfilling ≥ 75% of the appraisal criteria were categorized as high‐quality studies. Moreover, they were categorized as moderate quality if they met 50%–74% of the criteria, and the studies meeting ≤ 50% appraisal criteria were classified as low‐quality studies. This categorization aided in the interpretation of the findings of the included studies as per the potential risk of bias [15].

The qualities of the included studies are detailed in Table 2. According to Table 2, two out of three studies exhibited high quality levels as per the set criteria of the JBI critical appraisal tool. One of the included studies was of moderate–high quality. A detailed breakdown of the quality assessment of the included studies is provided below (Table 2).

**TABLE 2:** Quality assessment of the included studies by the JBI critical appraisal tool.

S. no.	Study	Study design	Randomization explained	Blinding techniques used	Presence of control group	Outcome measures clearly defined	Statistical analysis appropriate	Overall quality
1.	Hunsrisakhun et al. [16]	RCT	Yes	Double‐blind	Yes	Yes	Yes	High
2.	Parry et al. [17]	RCT	Yes	Yes	Yes	Yes	Yes	High
3.	Andrea et al. [18]	RCT	Yes	Not specified	Yes	Yes	Yes	Moderate–high

### 3.2. Main Findings

After completing the screening process, three studies were finalized for inclusion in this systematic review. The included studies were three randomized trials. The findings related to the effects of oral hygiene interventions (mouthwashes, rinses, saltwater, and specialized toothpastes) on the oral health of pregnant women.

Hunsrisakhun et al. conducted a 3‐month double‐blinded RCT among pregnant Thai women. These women were falling in the age bracket of 15–40 years, between the gestation period of 12–18 weeks, with at least 20 natural teeth in their oral cavities, with no gross dental problems or any other comorbidities/underlying systemic diseases. This study was conducted to evaluate the effectiveness of alcohol‐free mouthwash containing essential oils with 0.05% fluoride content (LISTERINE Natural Green Tea, Johnson and Johnson Consumer, USA) and alcohol‐free mouthwash with 0.05% fluoride only at the baseline period, and at 2 weeks, and 3 months postbaseline period, respectively. The sample size was calculated at 154 at a power of 90% and a difference of 0.21. A baseline examination was done using a self‐administered questionnaire, followed by an oral examination. A content‐validated, self‐administered questionnaire based on 29 sociodemographic items, 7 medical‐ and dental‐related questions, and 13 oral hygiene practice–related questions. In the oral examination, plaque index, gingival status, and tongue cleanliness were assessed. Prophylactic treatment was done in the selected participants to remove plaque and supragingival calculus. The participants were also guided to brush their teeth for at least 2 min twice a day. They were also given a toothbrush and a dentifrice containing 1000 ppm fluoride. Then, they were randomly allocated to intervention and control groups. After the dropouts from 154 participants, 140 were pregnant women (average age ± 26.8 years). There was no significant difference in the effects of alcohol‐free mouthwash containing essential oils with 0.05% fluoride content and alcohol‐free mouthwash with 0.05% fluoride only at the baseline period, 2 weeks, and 3 months postbaseline. Hence, mouthwash with alcohol‐free mouthwash containing essential oils with 0.05% fluoride content will have the same effects as that of alcohol‐free mouthwash with 0.05% fluoride only [16].

Botera et al. conducted a randomized clinical trial, with two parallel arms, among at least 4‐month pregnant women. These women were having at least Stage 1 Grade B of periodontitis and were falling in the age bracket of 18–35 years, regarding the use of paraprobiotic‐based toothpaste and paraprobiotic‐based mousse. The participants were divided into two groups. The paraprobiotic‐based toothpaste + mousse (Biorepair Peribioma line) was provided to the test group, while the control group was given only the toothpaste for 6 months. The 4 time points for the data collection were baseline (T0), 1 month (T1), 3 months (T2), and 6 months (T3). Significant effects were noted on bleeding on probing and plaque control when both of the products mentioned above were used in combination. No significant change was noted when the paraprobiotic‐based toothpaste was used alone. Overall, the recession was also noted to be reduced with these two products used in combination. Moreover, parabiotics were labeled safe during pregnancy since no adverse effects were reported as such [18].

Parry et al. conducted a clinical trial evaluating an intensive protocol used in maintaining oral hygiene with the utilization of antibacterial toothpaste and a nonalcoholic mouth rinse with antimicrobial properties among pregnant women. The study determined the oral health status of pregnant women and its impact on pregnancy outcomes, making it particularly relevant for determining its character of specific hygiene agents required during pregnancy. Following proper oral hygiene care will ultimately decrease the probability of preterm birth among pregnant women with gingivitis and other oral health challenges. Improvement in their oral hygiene by using antibacterial toothpaste and mouthwash may decrease their risk of acquiring complications during pregnancy. This study provided a review of active ingredients being used in mouthwashes and toothpastes, including CPC and fluoride compounds that are commonly used for pregnant women with periodontal concerns. The study included safety, efficacy, and the mechanism of action for several agents, supporting clinical decision‐making when treating pregnant patients [17].

## 4. Discussion

This systematic review focuses on the effectiveness of various oral hygiene maintenance agents, such as mouthwashes, rinses, and toothpastes, particularly for pregnant women. The included studies indicate notable improvements in the oral health status of the targeted populations when using these agents. Gingival health improved with plaque control and reduced inflammation. As Hunsrisakhun et al. stated in 2020, alcohol‐free mouthwashes containing fluoride and essential oils were as efficacious in plaque control as formulations containing fluoride only among women in Thailand [16]. Moreover, Andrea et al. in 2024 found that paraprobiotic‐based toothpaste and mousse, when used together, improve bleeding on probing and reduce gingival recession in women with early‐stage periodontitis. These findings were in coherence with the study by Jiang et al. in 2015, who stated that the use of mouthwash during pregnancy had positive effects on pregnant women as well as their offspring. The offspring had increased birth weight and reduced preterm deliveries [19].

Moreover, saltwater rinses were documented as the most effective and easily available intervention to maintain oral hygiene. Collins et al. compared the anti‐inflammatory effects of saltwater and 0.12% chlorhexidine rinses [13]. Ballini et al. further supported these results. In this study, the efficacy of sea salt and xylitol‐based rinses was found to reduce plaque and improve gingival health. These agents were found to be low‐cost alternatives [20]. This review only considered the efficacy, while previous research focused on the behavioral and systemic aspects as well [21, 22].

In previous literature by Bobetsis et al. [1], Xiong et al. [23], and Figuero [24], it has been noted that periodontal diseases have adverse effects on pregnancy outcomes, which is in coherence with our study [1, 23, 24]. In one of the studies by Xolboeva et al., therapeutic measures were proposed based on the scientific knowledge available through publications to improve pregnancy outcomes and decrease the prevalence of dental caries among pregnant women. This study was consistent with our study, in which the effects of different interventional methods were noted among pregnant women [3]. Previous studies by Salama et al. [25], Wu et al. [26], and Muthiani et al. [27] have shown that scaling along with root planning, combined with the use of mouthwash, will lower the preterm birth and low birth weight [25–27]. This study only considered mouthwashes and toothpastes as general terms and did not consider their compositions in detail. Also, this study only included pregnant women. In contrast to this, literature is available comparing different compositions used in mouthwashes and considering the patients or the general population, irrespective of gender [28, 29].

This study only included the effects of different mouthwashes, saltwater, or toothpastes. No risk factors were noted as such, while there are studies considering the risk factors associated with age among pregnant women [30]. Andrea et al. stated in one of the pilot studies that the efficacy of mouthwash, sea salt, or xylitol was found to be effective in maintaining oral health. This finding is in coherence with the present study [20].

Ayamolowo et al. reviewed the oral health behaviors among pregnant women, focusing on the use of toothpastes, practices to rinse, and dental hygiene. It included behavioral practices and challenges faced by pregnant women to maintain their oral hygiene during this critical period. This article offers insights into populations by aligning with the preventive and behavioral aspects [31]. However, in this study, the behavioral aspect was not included as such. It is further recommended to consider this aspect in future studies, as it plays a very important role in pregnancy.

Conjointly, these findings conclude that oral hygiene interventions, along with behavioral education within the cultural domains, can improve the pregnancy‐related oral health outcomes.

## 5. Conclusion

This systematic review was conducted among pregnant women. It concludes that practicing oral hygiene interventions, including saltwater rinses, fluoridated mouthwashes, and toothpastes, can significantly improve the oral health of pregnant women. These agents decrease plaque accumulation and improve gingival health, which are particularly important given the high risk pregnant women face. Future research on oral hygiene interventions among pregnant women is recommended, considering behavioral and cultural factors and broader populations. Moreover, given the limited available data on oral hygiene interventions among pregnant women, further research on this topic is also recommended.

### 5.1. Clinical Relevance

• Scientific rationale for study
   Pregnant women are at an amplified risk for oral health problems. Common oral care interventions, for example, toothpastes, mouthwash, saltwater, and rinses, can maintain oral health and possibly mitigate risks to pregnancy outcomes.
• Principal findings
  - Improved oral health status of women.
  - The impact of mouthwash, rinses, saltwater, and toothpastes on reducing gingivitis, plaque, and other oral health issues.
• Practical implications
  - Inform and guide pregnant women and healthcare providers about the benefits of using mouthwash, rinses, saltwater, and toothpastes for maintaining oral health.
  - Help in developing evidence‐based guidelines for oral healthcare during pregnancy.

**Nomenclature**
RCT: Randomized controlled trial
USA: United States of America
CPC: Cetylpyridinium chloride
JBI: Joanna Briggs Institute
PRISMA: Preferred Reporting Items for Systematic reviews and Meta‐Analyses

## Author Contributions

Conceptualization, M.M.J.; methodology, M.M.J., M.S., K.N., U.H.J., M.M.M., N.U.H., S.Z., S.A., S.I., and M.F.U.; validation, M.M.J., M.S., K.N., U.H.J., M.M.M., and M.M.J.; formal analysis, M.M.J., N.U.H., M.M.J., and M.F.U.; investigation, M.M.J., M.S., S.A., S.I., S.Z., and N.U.H.; writing–original draft preparation, M.M.J., U.H.J., M.M.M., N.U.H., S.Z., S.A., M.F.U., and S.I.; writing–review and editing, M.M.J., M.S., U.H.J., N.U.H., S.A., M.F.U., S.I., and M.M.J.; supervision, M.M.J. and M.S.

## Funding

The article processing charges (APC) will be covered by the University of Buckingham, Buckingham, United Kingdom.

## Disclosure

All authors have read and agreed to the published version of the manuscript.

## Ethics Statement

The authors have nothing to report.

## Conflicts of Interest

The authors declare no conflicts of interest.

## Data Availability

The data will be provided on request by the corresponding author.
