# Correction to “Effectiveness of Mouthwash, Rinses, Salt Water, and Toothpastes on the Oral Health of Pregnant Women: A Systematic Review: Pregnancy and Oral Health”

**DOI:** 10.1155/ghe3/9754054

**Published:** 2026-08-20

M. M. Javaid, M. Sarfraz, K. Noreen, et al., “Effectiveness of Mouthwash, Rinses, Salt Water, and Toothpastes on the Oral Health of Pregnant Women: A Systematic Review: Pregnancy and Oral Health,” *Global Health, Epidemiology and Genomics* 2026 (2026): 2627524, https://doi.org/10.1155/ghe3/2627524.

In the article titled “Effectiveness of Mouthwash, Rinses, Salt Water, and Toothpastes on the Oral Health of Pregnant Women: A Systematic Review: Pregnancy and Oral Health,” an affiliation for Muhammad Mohsin Javaid was inadvertently omitted. The missing affiliation has now been added as Affiliation 2, and the author affiliations have been updated accordingly.

Muhammad Mohsin Javaid^1,2^ Mariyum Sarfraz^1^ Khola Noreen^3^ Ume Habiba Johar^1^ Muhammad Mutahir Mehdi^4^ Najam Ul Hassan^5^ Shumaila Zofeen^6^ Shazia Iqbal^7^ Shahzad Ahmad^7,8^ Muhammad Farooq Umer^9^

^1^School of Public Health, Health Services Academy, Islamabad, Pakistan

^2^Department of Community and Preventive Dentistry, School of Dentistry, Shaheed Zulfiqar Ali Bhutto Medical University Islamabad, Pakistan

^3^Department of Community Medicine, Rawalpindi Medical University, Rawalpindi, Pakistan

^4^Department of Oral Biology, Nishtar Institute of Dentistry, Multan, Pakistan

^5^Department of Orthodontics and Dentofacial Orthopedics, School of Dentistry, Shaheed Zulfiqar Ali Bhutto Medical University, Islamabad, Pakistan

^6^School of Public Health, Xi’an Jiaotong University, Xi’an, Shaanxi, China

^7^Faculty of Medicine and Health Sciences, The University of Buckingham, Buckingham, UK

^8^IMBB, The University of Lahore, Lahore, Pakistan

^9^Department of Preventive Dental Sciences, College of Dentistry, King Faisal University, Hofuf 31982, Al Ahsa, Saudi Arabia

We apologize for this error.

